# Comment on Zheng et al. Association between Promoter Methylation of Gene ERCC3 and Benzene Hematotoxicity. *Int. J. Environ. Res. Public Health* 2017, *14*, 921

**DOI:** 10.3390/ijerph14111393

**Published:** 2017-11-16

**Authors:** Hanns Moshammer, Michael Poteser

**Affiliations:** Department of Environmental Health, Medical University of Vienna, ZPH, Kinderspitalgasse 15, 1090 Vienna, Austria; Michael.poteser@meduniwien.ac.at

**Keywords:** benzene, leukemia, hematotoxicity, epigenetics

## 1. Introduction

Benzene is an established carcinogenic substance [1,2]. Benzene displays rather low acute toxicity in various animal species [1], and benzene itself is not mutagenic in the classical bacterial tests. In mammal cell systems and after metabolic activation it is genotoxic due to oxidation mediated by Cytochrome P450 ([2] and references therein]). Although the International Agency for Research on Cancer (IARC) [2] provides a lengthy discussion on the mechanisms and tries to explain why genotoxicity in men is almost exclusively observed in the hemopoetic system, it still is surprising that other organs with high metabolic activity like the liver do not seem to be affected. Some epidemiological studies indicate also other target organs [3], but the findings are not as clear as with leukemia.

Indeed, there is a need for more complex explanation of causative pathways leading to leukemia targeting several pathways, including epigenetics [4]. Zheng et al. [5] report about epigenetic effects of benzene exposure and propose that the promoter region of the ERCC3 gene plays an important role in benzene hematoxicity.

## 2. Findings by Zheng et al.

Zheng et al. [5] examined 76 benzene-exposed workers and 24 unexposed controls. In an earlier study of the same participants [6], they found that the average methylation level in the promoter region of the ERCC3 gene was higher in the exposed workers. In the current study they examined specific sites with a cytosine-guanine sequence (CpG units) of that region and found the most significant effects in two CpG units 43 bp upstream and 99 bp downstream of the transcription start site (TTS). In all of the 10 CpG units examined the difference in percent of methylation between exposed and controls was between 0.15 and 1.83 percentage points. The two significant differences amounted to 0.96 and 1.66 percentage points.

In this study the authors also demonstrated a negative association between methylation of the CpG unit 43 bp upstream if the TTS and neutrophil counts among the subgroup of exposed workers. In their previous paper they had found lower neutrophil and total white blood cell counts in the exposed workers compared to controls.

## 3. Discussion

ERCC3 is involved in nucleotide excision repair and methylation of its promoter region, and likely reduces the transcription of the gene and hence the activity of the enzyme. It is conceivable that this plays a role in the genotoxic effects pathway of benzene. However, is it likely causal for leukopenia? The paper by Zheng et al. [5] shows intriguing associations that deserve further investigations. The question remains as to which type of investigations is called for. Let us consider the two findings of the paper separately.

Benzene causes epigenetic changes in many parts of the chromosomes including hypo- and hypermethylation, as reported by Zheng et al. [5] and McHale et al. [4]. These epigenetic changes do lead to genetic instability. Is the observed methylation pattern specific for benzene exposure and for blood cells, or is it the result of a more general reaction of different types of cells towards a broader range of toxic stressors?

Hypermethylation of the promoter region of ERCC3 is a plausible key factor, and it is likely that certain CpG units are more important than others. Further research will show if the two units that proved to be significantly affected by benzene exposure are really the most relevant. The difference in methylation was not larger in these two units than in the others. The difference of the other units was not significant because the background noise was larger. However, as other unknown factors are also affecting methylation, this does not mean that the effect of benzene exposure is not clinically relevant. A clearer picture could be found in in vitro studies where benzene effects could be studied without interference from uncontrolled other exposures.

Higher methylation was associated with lower neutrophil numbers, but this association was only visible in the group of exposed workers. If a higher methylation rate in that specific CpG unit was causally related to reduced cell counts, the association would have been visible irrespective of exposure status. Since the association is only seen in the exposed group it seems more likely that the hypermethylation only serves as a marker of exposure and not the hypermethylation per se, but the exposure (through another, unknown pathway) causes the reduction of neutrophils. Indeed, it seems more plausible that a reduced activity of a nucleotide excision repair enzyme is linked to leukemia rather than neutropenia.

Interestingly, the group of workers exposed to benzene consisted of two subgroups: Forty-one workers had experienced an episode of benzene poisoning approximately 20 years before the present study and had not been exposed after that episode. The other subgroup consisted of 35 healthy workers that were still working and experiencing exposure, and who, on average, had encountered the same cumulative exposure (>100 ppm-years). Epigenetic changes are long-lasting, but not necessarily permanent. If hypermethylation is (causally) associated with neutrophil counts the patients with acute benzene poisoning and severe leukopenia would also show the most severe hypermethylation. It would be interesting to study hypermethylation patterns in a longitudinal manner starting after an episode of toxic neutropenia. In the current study the once-poisoned subjects had recovered from that episode and their cell counts did not differ substantially from the other sub-group.

## 4. Conclusions

Epigenetic changes due to toxic exposures, not least also due to benzene, deserve further studies. These studies should combine longitudinal epidemiological and mechanistically more informative in vitro methods. The current paper [5] provides intriguing new insights but warrants confirmation and thorough interpretation. A statistical association could indicate a causal relationship. The epigenetic changes could be important stepping stones on the causal pathway to hematological pathologies. However, at the current stage of knowledge it is equally conceivable that these findings are only markers of exposure without inherent pathological importance.
